# The effect of dobutamine in sepsis: a propensity score matched analysis

**DOI:** 10.1186/s12879-021-06852-8

**Published:** 2021-11-11

**Authors:** Youfeng Zhu, Haiyan Yin, Rui Zhang, Xiaoling Ye, Jianrui Wei

**Affiliations:** 1 Department of Intensive Care Unit, Guangzhou Red Cross Hospital, Medical College, Jinan University, Guangdong 510220 Guangzhou, China; 2 Department of Critical Care Medicine, The First Affiliated Hospital of Jinan University, Guangzhou, 510220 Guangdong China; 3 Guangzhou Women and Children’s Medical Center, No. 9 Jinsui Road, Guangzhou, 510220 Guangdong China

**Keywords:** Dobutamine, Sepsis, Propensity score matched analysis, Hospital mortality

## Abstract

**Background:**

The use of dobutamine in patients with sepsis is questionable currently. As the benefit of dobutamine in septic patients is unclear, we aimed to evaluate whether the use of dobutamine was associated with decreased hospital mortality in sepsis patients.

**Methods:**

Based on the analysis of MIMIC III public database, we performed a big-data, real world study. According to the use of dobutamine or not, patients were categorized as the dobutamine group or non dobutamine group.We used propensity score matched (PSM) analysis to adjust for confoundings. The primary outcome was hospital mortality.

**Results:**

In the present study, after screening 38,605 patients, 2826 patients with sepsis were included. 121 patients were in dobutamine group and 2165 patients were in non dobutamine group. Compared with patients in non-dobutamine group, patients in dobutamine group had a lower MAP, higher HR, higher RR, higher severity of illness scores. 72 of 121 patients (59.5%) in the dobutamine group and 754 of 2165 patients (34.8%) in the non-dobutamine group died in the hospital, which resulted in a significant between-group difference (OR 1.56, 95% CI 1.01–2.40; P = 0.000). For the secondary outcomes, patients in dobutamine group received more MV use, more renal replacement therapy use, had longer ICU stay durations and more cardiac arrhythmias than those in non-dobutamine group. After adjusting for confoundings between groups by PSM analysis, hospital mortality was consistently higher in dobutamine group than that in non-dobutamine group (60.2% vs. 49.4%, OR 1.55, 95% CI 1.01–2.37; P = 0.044).

**Conclusions:**

Among patients with sepsis, our study showed that the use of dobutamine was not associated with decreased hospital mortality. Further large scale, randomized controlled studies are warrented to confirm our findings.

**Supplementary Information:**

The online version contains supplementary material available at 10.1186/s12879-021-06852-8.

## Background

Dobutamine is recommended for septic patients who have myocardial depression according to surviving sepsis campaign guidelines, especially in those with evidence of persistent hypoperfusion, despite sufficient fluid resuscitation and the administration of vasopressor drugs [1]. However, there is uncertainty whether it has robust effects on patient centered outcomes such as mortality. The data supporting the use of dobutamine are mainly physiologic, which may improve some variables of perfusion and ameliorate hemodynamics, such as improving central venous oxygen saturation and decreasing lactate levels [1].

So far, no randomized controlled trials have performed to evaluate whether there are significant differences between dobutamine and placebo on clinical outcomes in septic patients. In the three EGDT validation studies (ProMISe, ProCESS and ARISE studies), the use of dobutamine was more frequent in the EGDT group than in the control group (ProMISe study 8.0 vs. 1.1%, respectively, p < 0.001;ProCESS study 8.0 vs. 1.1%, respectively, p < 0.001; and ARISE study 15.4 vs. 2.6%, respectively, p < 0.0001), but the mortality outcomes were similar in both group [2–4]. And the adverse effects with the use of dobutamine were not detected in the previous studies.

Dobutamine increases myocardial oxygen consumption, raises myocardial work, reduces cardiac efficiency, although it can increase cardiac index [5, 6]. Furthermore, some studies reported the use of dobutamine was cardiotoxic, which might induce eosinophilic cardiomyopathy or takotsubo cardiomyopathy [7–9]. Hence, the use of dobutamine in patients with sepsis is questionable currently. As the benefit of dobutamine in septic patients is unclear so far, in this study, we aimed to evaluate whether the use of dobutamine was associated with decreased hospital mortality in sepsis patients.

## Methods

We performed this study in accordance with the STrengthening the Reporting of OBservational studies in Epidemiology (STROBE) statement [10]. This was a big-data, real world study based on the third edition of the Medical Information Mart in Critical Care (MIMICIII) database, which was developed and maintained by the Laboratory for Computational Physiology at MIT [11, 12] The MIMICIII database included longitudinal data on 38,605 patients who were admitted to the ICU of Beth Israel Deaconess Medical Center from 2002 to 2011 for a total of 53,423 distinct admissions. As the present study was based on the analysis of the MIMICIII public database, ethical review and informed consent were waived.

### Patients

We screened the discharge diagnosis of patients in MIMIC III database by ICD 9 and ICD 10 codes. Adult patients (age ≥ 18 years) who had a discharge diagnosis of sepsis, severe sepsis or septic shock and were admitted to the ICU from 2002 to 2011 in the MIMICIII database were screened for inclusion.

Patients who were under age 18, were pregnant, had obstructive hypertrophic cardiomyopathy, or had no detailed demographic information, were excluded.

The patients who used dobutamine during ICU stay were categorized as the dobutamine group, with the remaining patients were categorized as the non dobutamine group.

### Sepsis diagnosis

The definitions and diagnositic criteria for sepsis, severe sepsis and septic shock were unchanged between 2002 and 2011, according to the Surviving Sepsis Campaign Guidelines [1, 13, 14].

### Data collection

In this study, only each patient’s first ICU admission data were included. The following demographic data and admission information were collected: age, gender, weight, height, body mass index (BMI), mean arterial pressure (MAP), heart rate (HR), respiratory rate (RR), temperature, Simplified Acute Physiology Score-I (SAPS-I), Simplified Acute Physiology Score-II (SAPS-II), Sequential Organ Failure Assessment (SOFA), Elixhauser comorbidity index (ECI), admission type (emergency or elective), sepsis type (sepsis, severe sepsis, septic shock).

Additionally, data regarding the use of mechanical ventilation (MV) or renal replacement therapy (RRT) within the first day of ICU admission were collected.

Microbiology events were recorded and the following laboratory results were also collected: lactate value at admission of ICU,the maximum level of blood glucose level, the minimum level of glucose level.

### Comorbidities

We recorded the chronic comorbidities of our study cohort. The MIMICIII database contains over 15,693 different diagnoses classified by ICD 9 and ICD 10 codes. For describing chronic diseases more concisely, we used Elixhauser’s comorbidity classification [14] according to an algorithm provided by the authors of the MIMICIII database [15]. Chronic diseases can effectively be reflected by the Elixhauser comorbidity classification, and they have been validated for both ICD 9 and ICD 10 codes [16, 17].

### Primary and secondary outcomes

The primary outcome was hospital mortality rate, and the secondary outcomes included number of patients who received MV or RRT during their hospital stay, number of patients received norepinephrine use, length of ICU stay and length of hospital stay, cardiac arrthymias. Cardiac arrhythmias data was collected through discharge diagnosis according to the ICD 9 and ICD 10 codes.

### Statistical analysis

The details of the data screening strategies used are shown in the Additional file 1. Other source codes for our analyses, which were provided by the authors of the MIMICIII database, can be found at GitHub [16, 18]. Categorical variables including demographic data, admission information, and interventions were shown as frequencies, and continuous variables including vital signs and laboratory parameters were presented as mean ± standard deviation (SD) or median with interquartile range (25, 75%). We used the analysis of variance or non-parametric tests to analyze continuous variables as appropriate. Categorical variables were analyzed using Fisher’s exact test or Pearson’s chi-square test. We used odds ratios (ORs) and 95% confidence intervals (CIs) for outcome analysis between groups.

### Propensity score matched analysis

For the primary outcome of hospital mortality, in order to ensure the robustness of our results, we used the propensity score matched (PSM) method to adjust and balance the influence of confounding factors between groups. The variables included in the PSM analysis for matching were as following: age, gender, BMI, MAP, SOFA score, SAPS-I score, SAPS-II score, ECI, sepsis type, admission type, congestive heart failure, coagulopathy disease, lactate level and Glucose-max, norepinephrine use before dobutamine administration, urine volume in the first day of ICU admission. Demographic data and admission information were balanced. Each dobutamine group patient was matched with no dobutamine patients at a proportion of 1:3 with the closest propensity score. The matching caliper was 0.2. PSM analysis required no missing datapoint, so we excluded recorded with data missing. As the data of lactate values are missing a moderate amount, and excluding any patient with a single missing lactate value datapoint will lead to a significant selection bias. Hence, multiple imputation strategies are used for missing lactate values to overcome this deficit in the PSM analysis.

### Subgroup analysis

We also performed subgroup analyses to further investigate whether the primary outcome was different among subgroups. According to the previous studies [19–21] that revealed the risk factors of mortality in sepsis patients and reported the mortality rates might be different among these subgroups, we included the following subgroups: age (< 60 years; ≥ 60 years), BMI (≥ 28 kg/m^2^; < 28 kg/m^2^) and gender.

We used PostgreSQL 10.0 software (University of California, Berkeley, California, USA) and Navicat premium 12.0 software (premiumSoft Cybertech Ltd, Kowloon, Hong Kong, China) for database management and data retrieval and screening; R software (version 2.15.x, GNU project) and SPSS 22.0 software (IBM Corp., Armonk, NY, USA) were used for statistical analysis. A two sided P < 0.05 was statistical significance.

## Results

**Fig. 1:**
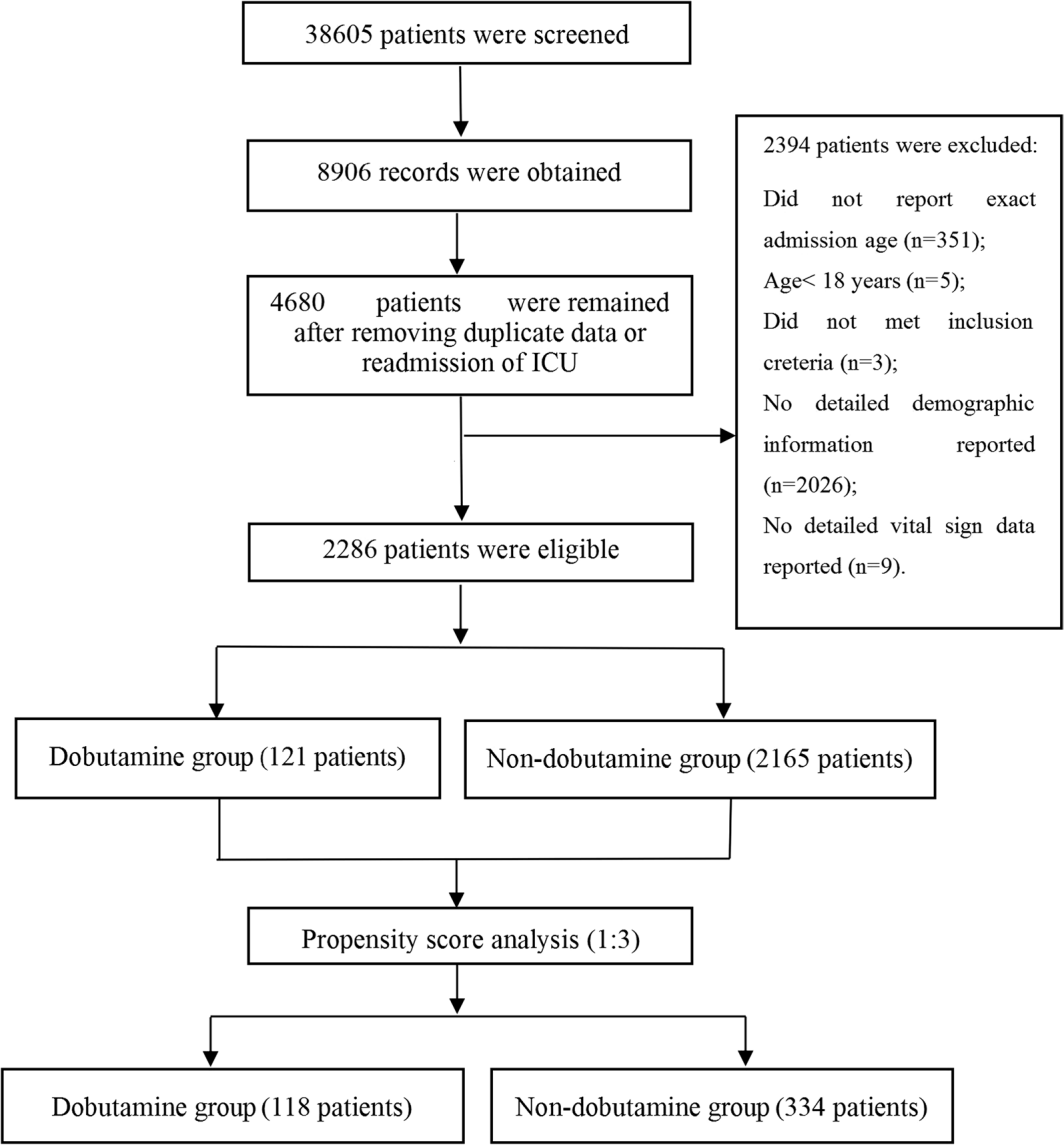
Study screening and selection process. *ICU* intensive care unit

Initially, 38,605 patients in the MIMIC III database were screened for eligibility, and 8906 records were included. After removing duplicate records or readmissions to the ICU, 4680 sepsis patients were left. We further screened the demographic data and admission information, and 2394 patients were removed due to information insufficient. Ultimately, 2286 patients were included in this study (Fig. 1). And according to the usage of dobutamine or not, patients were divided into the dobutamine group or the non-dobutamine group. In total, there were 121 patients in the dobutamine group and 2165 patients in the non-dobutamine group.

The clinical characteristics and laboratory results of the included patients are shown in Table 1. Compared to patients in non-dobutamine group, patients in dobutamine group had a significant lower MAP (46.44 ± 15.51 mmHg vs. 51.63 ± 14.01 mmHg, p < 0.001), higher HR (118.60 ± 23.46 beats/min vs.112.71 ± 22.97 beats/min, p = 0.006), higher RR (31.78 ± 8.00 breaths/min vs.30.23 ± 7.11 breaths/min, p = 0.020), higher SOFA score (9.91 ± 3.73 vs. 7.22 ± 4.00, p < 0.001), higher SOFA-cardiovascular score (3.23 ± 1.20 vs. 2.26 ± 1.45, p < 0.001), SAPS-I score (24.86 ± 5.64 vs. 21.46 ± 5.68, p = 0.000) and SAPS-II score (56.59 ± 14.83 vs. 46.76 ± 16.66, p < 0.001). More patients in dobutamine group received mechanical ventilation at the first day of ICU admission than those in non-dobutamine group (95/121 patients vs.1128/2165 patients, p = 0.000). Furthermore, more patients in dobutamine group received norepinephrine (90.9% vs.58.6%, p = 0.000) and the norepinephrine doses were higher in dobutamine group than those in non-dobutamine group (79.89 ± 96.44 mg vs. 58.30 ± 82.56 mg, p < 0.001). The volumes of fluid administered were similar between both groups in the first day of ICU admission. However, the urine volumes in the first day were less in dobutamine group (1083.63 ± 1005.80 vs. 1565.61 ± 1333.18 ml, p < 0.001) (Table 1).

**Table 1:** Baseline demographic data and clinical characteristics of patients included in the study

Covariate	Original patients (before matching)			Missing data (%)
	Non-dobutamine group (N = 2165)	Dobutamine group (N = 121)	P value	
Age (years)	66.63 ± 17.06	68.57 ± 16.44	0.222	0.0
Gender (Male), n (%)	1208 (55.8%)	74 (61.2%)	0.248	0.0
Height (cm)	168.76 ± 11.00	168.61 ± 9.68	0.883	0.0
Weight (kg)	82.65 ± 27.00	83.26 ± 21.95	0.808	0.0
BMI (kg/m [2])	29.00 ± 9.00	29.13 ± 6.72	0.871	0.0
Temperature (℃)	37.71 ± 1.04	37.87 ± 1.11	0.108	0.4
MAP (mmHg)	51.63 ± 14.01	46.44 ± 15.51	0.000	0.0
HR (beats min^−1^)	112.71 ± 22.97	118.60 ± 23.46	0.006	0.0
RR (min^−1^)	30.23 ± 7.11	31.78 ± 8.00	0.020	0.0
SOFA score	7.22 ± 4.00	9.91 ± 3.73	0.000	0.0
SOFA-cardiovascular score	2.26 ± 1.45	3.23 ± 1.20	0.000	0.0
SAPS-I score	21.46 ± 5.68	24.86 ± 5.64	0.000	0.0
SAPS-II score	46.76 ± 16.66	56.59 ± 14.83	0.000	0.0
ECI	10.36 ± 7.91	11.06 ± 7.92	0.342	0.0
Norepinephrine dose (mg)	58.30 ± 82.56	79.89 ± 96.44	0.004	0.0
Norepinephrine use	1269 (58.6%)	110 (90.9%)	0.000	0.0
Urine volume (ml, 1st day)	1565.61 ± 1333.18	1083.63 ± 1005.80	0.000	1.0
Input volume (ml, 1st day)	8209.26 ± 6142.67	9471.47 ± 7166.17	0.069	4.3
Microbiology, n (%)				
Positive (%)	1719 (79.4%)	99 (81.8%)	0.521	0.0
Negative	446 (20.6%)	22 (18.2%)		
Interventions, n (%)				
Renal replacement use (1st day)	170 (7.9%)	15 (12.4%)	0.074	0.0
Mechanical ventilation use (1st day)	1128 (52.1%)	95 (78.5%)	0.000	0.0
Comorbidities, n (%)				
Congestive heart failure	780 (36.0%)	75 (62.0%)	0.000	0.0
Pulmonary circulation disease	168 (7.8%)	10 (8.3%)	0.840	0.0
Renal failure	469 (21.7%)	23 (19.0%)	0.489	0.0
Diabetes	676 (31.2%)	44 (36.4%)	0.236	0.0
COPD	496 (22.9%)	33 (27.3%)	0.268	0.0
Coagulopathy	614 (28.4%)	53 (43.8%)	0.000	0.0
Fluid electrolyte disorder	1204 (55.6%)	66 (54.5%)	0.818	0.0
Admission type, n (%)				
Emergency	2106 (97.3%)	113 (93.4%)	0.014	0.0
Elective	59 (2.7%)	8 (6.6%)		0.0
Sepsis type, n (%)				
Sepsis	451 (20.8%)	7 (5.8%)	0.000	0.0
Severe sepsis	896 (41.4%)	50 (41.3%)		0.0
Septic shock	818 (37.8%)	64 (52.9%)		0.0
Laboratory Tests				
Lactate (mmol/L)	2.59 ± 2.19	3.50 ± 2.86	0.001	48.4
Glucose-min	106.90 ± 38.77	108.06 ± 48.17	0.753	0.5
Glucose-max	190.70 ± 94.38	227.50 ± 93.32	0.000	0.5

Data are mean ± SD, median (interquartile) or n (%). *SOFA* Sequential Organ Failure Assessment, ranging from 0 to 24, with higher scores indicating a greater degree of organ failure; *ECI* Elixhauser comorbidity index, and we used the modified vanWalraven Elixhauser comorbidity score in our study, which consists of 30 comorbidity diseases, range from − 19 to 89 points, with higher scores indicating a greater risk of hospital mortality, *MAP* mean arterial pressure; HR: heart rate, *RR* respiratory rate, *SAPS-I* simplified acute physiologic score-I, *SAPS-II* simplified acute physiologic score-II, *ICU* intensive care unit, *COPD* chronic obstructive pulmonary disease. The definitions and diagnositic criteria for sepsis, severe sepsis and septic shock were made according to the Surviving Sepsis Campaign Guidelines [1, 13, 14]

The culture positive rates of microbiology samples were similar between both groups (1719/2165 patients vs. 99/121 patients, p = 0.521) (Table 1). With regards to comorbidities, more patients in dobutamine group had congestive heart failure (75/121 patients vs. 780/2165 patients, p < 0.001) and coagulopathy disease (53/121 patients vs. 614/2165 patients, p < 0.001) than those in non-dobutamine group (Table 1).

### Primary outcome and PSM analysis

For the primary outcome of hospital mortality, 72 of 121 patients (59.5%) in the dobutamine group and 754 of 2165 patients (34.8%) in the non-dobutamine group died in the hospital, which resulted in a significant between-group difference (OR 1.56, 95% CI 1.01–2.40; P = 0.000) (Table 2).

**Table 2:** Comparison of primary and secondary outcomes

Outcomes n (%)	Original patients (before matching)				PSM adjusted patients (after matching)			
	Non-dobutamine group (N = 2165)	Dobutamine group (N = 121)	OR (95%CI)	P value	Non-dobutamine group (N = 334)	Dobutamine group (N = 118)	OR (95% CI)	P value
Hospital mortality	754 (34.8%)	72 (59.5%)	2.75 (1.89–4.00)	0.000	165 (49.4%)	71 (60.2%)	1.55 (1.01–2.37)	0.044
MV use	1312 (60.6%)	108 (89.3%)	5.40 (3.02–9.66)	0.000	268 (80.2%)	105 (89.0%)	1.99 (1.05–3.76)	0.032
RRT use	208 (9.6%)	28 (23.1%)	2.83 (1.81–4.43)	0.000	56 (16.8%)	27 (22.9%)	1.47 (0.88–2.47)	0.140
Norepinephrine free days	13.01 ± 13.74	11.47 ± 11.10	Na	0.255	12.68 ± 13.54	11.34. ± 11.20	Na	0.360
Cardiac arrhythmias	912 (42.1%)	68 (56.2%)	1.76 (1.22–2.55)	0.002	171 (51.2%)	67(56.8%)	1.25 (0.82–1.91)	0.297
Duration of ICU stay (days)	8.08 ± 9.97	11.77 ± 11.09	Na	0.000	10.20 ± 10.43	11.70 ± 11.18	Na	0.189
Duration of hospital stay (days)	14.98 ± 15.15	14.98 ± 12.22	Na	0.997	15.54 ± 14.22	14.79 ± 12.29	Na	0.611

*MV* mechanical ventilation, *RRT* renal replacement therapy

For the secondary outcomes, more patients in dobutamine group received MV use, RRT use, norepinephrine use than those in non-dobutamine group (Table 2). Compared with patients in non-dobutamine group, the duration of ICU stay was longer for patients in dobutamine group (Table 2).

For adverse events, more cardiac arrhythmias occured in dobutamine group than those in non-dobutamine group (Table 2).

In order to test the robustness of the primary and secondary outcomes, a PSM analysis (1:3) was performed. The baseline patients data included in the PSM analysis were shown in Table 3. The PSM analysis showed that results were consistent with hospital mortality and MV use (Table 2).

**Table 3:** Baseline demographic data and clinical characteristics of patients included in the PSM analysis

Covariate	Original patients (after matching)		
	Non-dobutamine group (N = 334)	Dobutamine group (N = 118)	P value
Age (years)	70.93 ± 19.89	68.48 ± 16.53	0.231
Gender (Male), n (%)	204 (61.1%)	71 (60.2%)	0.862
Height(cm)	168.78 ± 10.80	168.48 ± 9.70	0.787
Weight (kg)	82.08 ± 26.41	83.06 ± 22.16	0.719
BMI (kg/m^2^)	28.75 ± 8.59	29.10 ± 6.80	0.683
Temperature (℃)	37.63 ± 1.16	37.88. ± 1.12	0.044
MAP (mmHg)	47.38 ± 15.37	46.46 ± 15.69	0.579
HR (beats min^−1^)	115.41 ± 23.62	118.93 ± 23.28	0.163
RR (min^−1^)	30.69 ± 7.56	31.75 ± 8.09	0.199
SOFA score	9.66 ± 3.93	9.87 ± 3.73	0.606
SOFA-cardiovascular score	3.13 ± 1.25	3.21 ± 1.20	0.517
SAPS-I score	24.50 ± 5.69	24.83 ± 5.63	0.590
SAPS-II score	56.55 ± 16.63	56.39 ± 14.87	0.925
ECI	11.00 ± 7.47	11.21 ± 7.91	0.797
Norepinephrine dose (mg)	64.97 ± 90.54	75.78 ± 89.60	0.289
Norepinephrine use	305 (91.3%)	107 (90.7%)	0.833
Urine volume (ml, 1st day)	1169.60 ± 1123.08	1096.28 ± 1011.60	0.534
Input volume (ml, 1st day)	9500.05 ± 7096.23	9488.29 ± 7257.87	0.988
Microbiology, n (%)			
Positive (%)	275 (82.6%)	96 (81.4%)	0.764
Negative	58 (17.4%)	22(18.6%)	
Interventions, n (%)			
Renal replacement use (1st day)	41 (12.3%)	14 (11.9%)	0.907
Mechanical ventilation use (1st day)	244 (73.1%)	92 (78.0%)	0.294
Comorbidities, n (%)			
Congestive heart failure	204 (61.1%)	73 (61.9%)	0.880
Pulmonary circulation disease	21 (6.3%)	10 (8.5%)	0.419
Renal failure	77 (23.1%)	22 (18.6%)	0.319
Diabetes	112 (33.5%)	43 (36.4%)	0.567
COPD	81 (24.3%)	33 (28.0%)	0.424
Coagulopathy	137 (41.0%)	51 (43.2%)	0.676
Fluid electrolyte disorder	166 (49.7%)	66 (55.9%)	0.244
Admission type, n (%)			
Emergency	310 (92.8%)	110 (93.2%)	0.883
Elective	24 (7.2%)	8 (6.8%)	
Sepsis type, n (%)			
Sepsis	18 (5.4%)	7 (5.9%)	0.913
Severe sepsis	143 (42.8%)	48 (40.7%)	
Septic shock	173 (51.8%)	63 (53.4%)	
Laboratory tests			
Lactate (mmol/L)	3.01 ± 2.56	3.55 ± 2.49	0.047
Glucose-min	104.74 ± 40.59	108.08 ± 48.52	0.466
Glucose-max	211.77 ± 112.57	225.84 ± 93.33	0.224

Data are mean ± SD, median (interquartile) or n (%). *SOFA* Sequential Organ Failure Assessment, ranging from 0 to 24, with higher scores indicating a greater degree of organ failure, *ECI* Elixhauser comorbidity index, and we used the modified vanWalraven Elixhauser comorbidity score in our study, which consists of 30 comorbidity diseases, range from -19 to 89 points, with higher scores indicating a greater risk of hospital mortality, *MAP* mean arterial pressure, *HR* heart rate, *RR* respiratory rate, *SAPS-I* simplified acute physiologic score-I; *SAPS-II* simplified acute physiologic score-II, *ICU* intensive care unit, *COPD* chronic obstructive pulmonary disease. The definitions and diagnositic criteria for sepsis, severe sepsis and septic shock were made according to the Surviving Sepsis Campaign Guidelines [1, 13, 14]. No variable had a missing value

### Subgroup analyses for primary outcomes

**Fig. 2:**
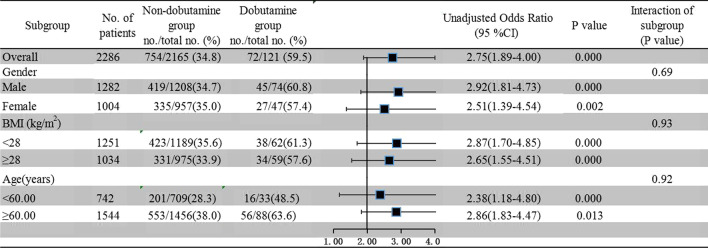
Subgroup analyses with regard to hospital mortality. *BMI* body mass index

Subgroup analyses with regards to hospital mortality according to gender, age (≥ 60 years, < 60 years), BMI (≥ 28 kg/m^2^, < 28 kg/m^2^) showed that there were no significant interactions between subgroups (Fig. 2).

## Discussion

In this retrospective study, a total of 2286 patients with sepsis were included. Compared with patients in non-dobutamine group, patients in dobutamine group had a lower MAP, higher HR, higher RR, higher severity of illness scores. Furthermore, more patients in dobutamine group received mechanical ventilation at the first day of ICU admission than those in non-dobutamine group. Despite these factors showed that dobutamine group patients were sicker, we found a signifcantly higher hospital mortality among patients who received dobutamine use, and the result was consistant after adjustment for confoundings by PSM analysis. Furthermore, more patients in dobutamine group received MV use, RRT use, had longer durations of ICU stay and more cardiac arrhymias than those in non-dobutamine group.

Dobutamine as an catecholamine working through the β1-adrenoceptor-Gα_s_ protein- adenylate cyclase- cAMP pathway, is recommended in septic patients with myocardial dysfunction [1]. However, the data supporting the use of dobutamine are mainly physiologic. So far, no randomized controlled trials have performed to evaluate whether there are significant differences between dobutamine and placebo on clinical outcomes in septic patients [1]. Further researches regarding the effects of dobutamine on patient centered outcomes such as mortality are warranted [22].

Our studies showed a signifcantly higher hospital mortality among dobutamine group than non dobutamine group, and the result was consistant after adjustment for confoundings by PSM analysis. Our findings was similar with a previous study by Wilkman and colleagues, in which patients received inotrope use had a higher 90-day mortality (42.5% vs. 23.9%, P < 0.001) and hospital mortality (18.5% vs. 33.8%, P < 0.001) than those without [23]. The hospital mortality was higher in our study, which might be due to patients in our study were older than those in Wilkman’s study (66.63 ± 17.06 years in non-dobutamine group and 68.57 ± 16.44 years in dobutamine group in our study, 53.3 ± 13.9 years in no inotropes group and 56.4 ± 14.6 years in inotropes group in Wilkman’s study). In addition, in our study, the overall mortality rate of sepsis patients was 36.1% (95% CI 34.2%-38.1%), which was similar with the rates reported in other large-scale studies [24, 25], demonstrating the high reliability of our study.

Furthermore, this study showed that patients in dobutamine group received more MV and RRT therapy, had longer durations of ICU stay and more cardiac arrhymas than those in non-dobutamine group. These results demonstrated that benefits of dobutamine use may have been overemphasized.

One explanation for our findings might be that effect of dobutamine as a positive inotrope is impaired in sepsis. Mari and colleagues found that the cardial responsiveness to dobutamine inotropic effect was significantly diminished in septic mice model compared with sham-operated controls [26]. They further found that this was related to upregulated phosphodiesterase 4D leading to plasma cAMP breakdown. Understanding the effect of phosphodiesterase 4D in regulating cardiac responsiveness to dobutamine may provide the potential of a PDE4D targeted therapy for sepsis patients with low cardiac output requiring inotropic support in future. Another explanation might be that the use of dobutamine was associated with adverse effect. A previous study showed that dobutamine also may dobutamine may promote inflammatory response by increasing circulating TNF-a levels in patients with septic shock [27]. The use of catecholamines could decrease metabolic efficiency and increase bacterial growth [6]. Furthermore, the use of dobutamine to increase oxygen delivery to a supernormal target did not improve mortality in critically ill patients [28, 29]. There are also some evidences that inotropic drugs are associated with worse outcomes in heart failure and cardiac surgery patients [30–32].

Some studies reported the adverse effects caused by inotropes, including myocardial oxygen consumption increase, higher incidence of arrhythmias and myocardial ischemia [7, 8, 33, 34]. In a randomized controlled trial, 516 patients with septic shock were randomly assigned to placebo group or levosimendan group; no difference was found in mortality. However, there was more tachyarrhythmias in levosimendan group than placebo group (absolute difference, 2.7%, 95% CI 0.1–5.3%) [35]. Our study also found that more cardiac arrhythmias occured in dobutamine group compared with non-dobutamine group.

The advantages of this study were that we performed a big data, large scale research based on the MIMIC III database, included patients with rigorous criteria, and performed subgroup and propensity score matched analysis to adjust for confoundings between the groups, which increased the reliability of the primary and secondary outcomes.

Our study has several limitations. First, our study was based on the analysis of MIMICIII database. Due to the retrospective nature of this study, there was unavoidable risk of bias. To decrease the influences of selection bias of more critically ill patients tending to receive dobutamine, our study adjusted for the baseline characteristics between non-dobutamine and dobutamine groups by propensity score matched method, and we further studied the primary outcomes through multiple subgroup analyses. Ultimately, the results were still consistent, which demonstrated that our results were reliable. Second, sepsis-induced cardiomyopathy (SIC) was reported in many studies which was characterised with left ventricular diastolic dysfunction and depressed ejection fraction. The incidence rate of SIC was high, approximately 24%-40% [36, 37]. Furthermore, some studies showed that diastolic dysfunction might be aggravated by catecholamines [38, 39]. Hence, some septic patients with diastolic dysfunction might account for a large proportion of patients who used dobutamine. And these patients might not benefit from dobutamine use. However, this aspects was not assessed in this study. Third, several hemodynamic variables as CVP, pulmonary capillary wedge pressure (PCWP) or cardiac index (CI) were not included in this study due to data insuffecient, which might influence the analysis of possible effects of dobutamine. However, a thorough physiologic and clinical examination variables (heart rate, temperature, respiratory rate, mean blood pressure) were included in our study. Furthermore, this study raised the concerns about dobutamine use in septic patients. Further large scale, randomized controlled studies are warrented to confirm our findings.

## Conclusion

Among patients with sepsis, our study showed that the use of dobutamine was not associated with decreased hospital mortality. The mechanism remains to be explored. Further large scale, randomized controlled studies are warrented to confirm our findings.

## Supplementary Information

**Additional file 1: Figure S1**. Distribution of propensity scores between treatment group (Dobutamine group) and control group (Non-dobutamine group) before and after match. **Figure S2**. The absolute standardized difference in Means before and after match. **Figure S3**. The density of propensity score between treatment group (Dobutamine group) and control group (Non-dobutamine group) before and after match. **Figure S4.** The distribution of standardized difference in Means before and after match. **Figure S5**. The propensity score of variables between treatment group (Dobutamine group) and control group (Non-dobutamine group) before and after match. Data Secreening Strategy

## Data Availability

The Datasets are available from the corresponding author on reasonable request. The details of the data screening strategies used are shown in the supplementary file. Other source codes for our analyses, which were provided by the authors of the MIMICIII database, can be found at GitHub (https://github.com/MIT-LCP/mimic-code).

## Abbreviations

BMI: Body mass index
SOFA: Sequential Organ Failure Assessment
ECI: Elixhauser comorbidity index
MAP: Mean arterial pressure
HR: Heart rate
RR: Respiratory rate
SAPS-I: Simplified acute physiologic score-I
SAPS-II: Simplified acute physiologic score-II
ICU: Intensive care unit
COPD: Chronic obstructive pulmonary disease
ORs: Odds ratios
CI: Confidence intervals
PSM: Propensity score matched analysis
