# Supplementary material for: The effect of dobutamine in sepsis: a propensity score matched analysis
Source: BMC Infect Dis. 2021 Nov 11;21:1151. doi: 10.1186/s12879-021-06852-8 (PMC8582172; doi:10.1186/s12879-021-06852-8)
Supplement: Supplementary file 1 — Additional file 1: Figure S1. Distribution of propensity scores between treatment group (Dobutamine group) and control group (Non-dobutamine group) before and after match. Figure S2. The absolute standardized difference in Means before and after match. Figure S3. The density of propensity score between treatment group (Dobutamine group) and control group (Non-dobutamine group) before and after match. Figure S4. The distribution of standardized difference in Means before and after match. Figure S5. The propensity score of variables between treatment group (Dobutamine group) and control group (Non-dobutamine group) before and after match. Data Secreening Strategy [file 12879_2021_6852_MOESM1_ESM.doc]

**The effect of dobutamine in sepsis: a propensity score matched analysis**

Youfeng Zhu^1&^*, Haiyan Yin^2&^, Rui Zhang^1^, Xiaoling Ye^1^ and Jianrui Wei^3^*

1.Department of Intensive Care Unit, Guangzhou Red Cross Hospital, Jinan University, Guangzhou 510220, Guangdong Province, China

2.Department of Critical Care Medicine, The First Affiliated Hospital of Jinan University, Guangzhou 510220, Guangdong Province, China

3. Department of Cardiology, Guangzhou Women and Children’s Medical Center, Guangzhou 510220, Guangdong Province, China

*Correspondence: Professor Jianrui Wei, M.D., No. 9 Jinsui Road, Guangzhou Women and Children’s Medical Center, Guangzhou 510220, Guangdong Province, China, +8618928900398, Jianruiw@163.com, fax +8602034403827.

Dr. Youfeng Zhu, Guangzhou Red Cross Hospital, Medical College, Jinan University, Guangzhou 510220, Guangdong Province, China,+8615813342900, 151276953@qq.com

Youfeng Zhu, M.D: 151276953@qq.com; Rui Zhang, M.D: 63281796@qq.com;

Haiyan Yin, M.D: yinhaiyan1867@126.com;

Xiaoling Ye, M.D:1718768452@qq.com

& These authors are co-first authors.

**Word counts:** 2612.

**Supplemental files**

**Figure S1 Distribution of propensity scores between treatment group (Dobutamine group) and control group (Non-dobutamine group) before and after match.**


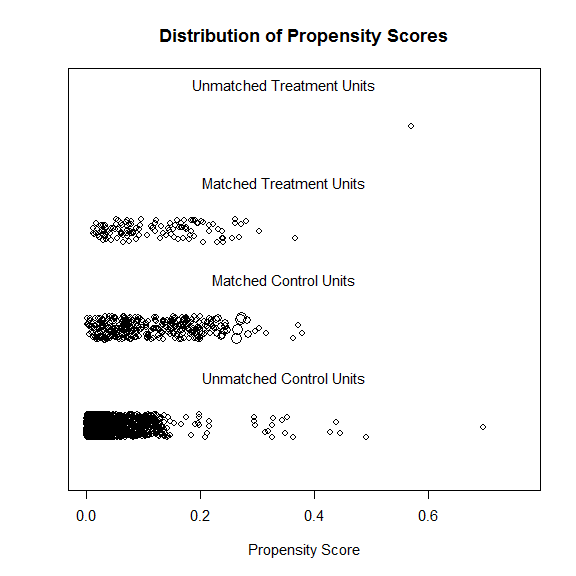


**Figure S2 The absolute standardized difference in Means before and after match.**


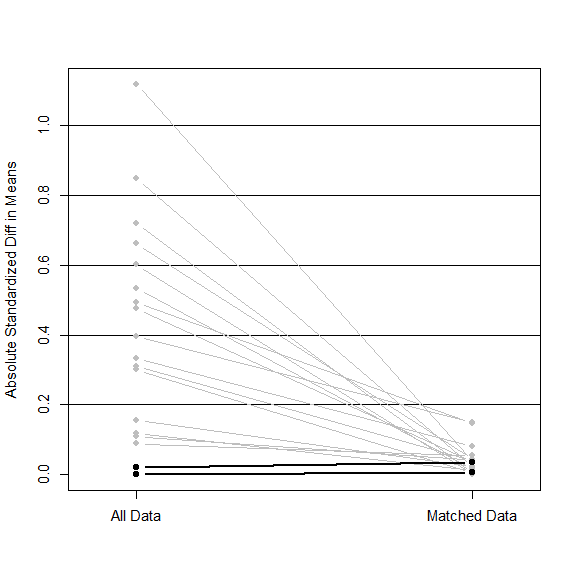


**Figure S3 The density of propensity score between treatment group (Dobutamine group) and control group (Non-dobutamine group) before and after match.**


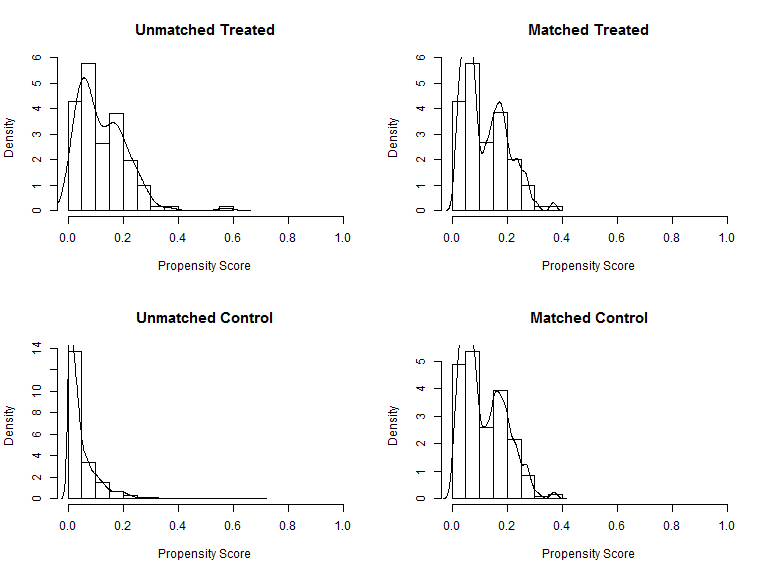


**Figure S4 The distribution of standardized difference in Means before and after match.**


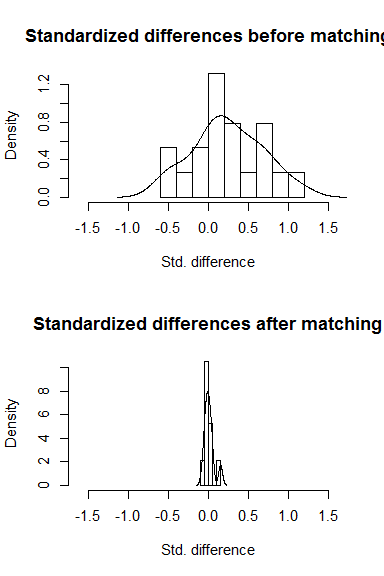


**Figure S5 The propensity score of variables between treatment group (Dobutamine group) and control group (Non-dobutamine group) before and after match.**


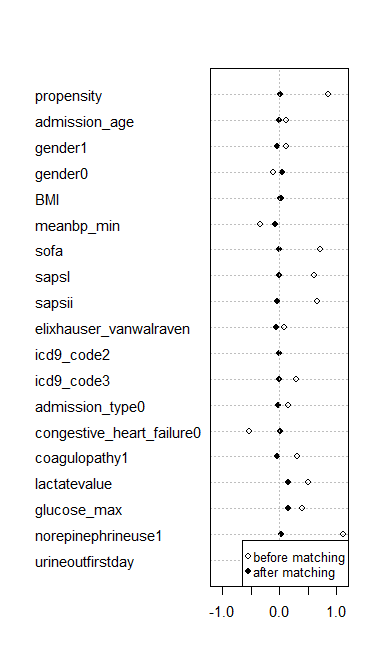


**Data Secreening Strategy**

DROP MATERIALIZED VIEW IF EXISTS unititled1 CASCADE;

CREATE MATERIALIZED VIEW unititled1 AS

SELECT unititled.subject_id, unititled.icd9_code, unititledtitled.icustay_id, unititled.los_hospital, unititled.los_icu, unititled.admission_age, unititled.hospital_expire, unititled.hadm_id, unititled.gender, unititled.admittime, unititled.dischtime, unititled.admission_type, heightweight.height_first, heightweight.weight_first FROM unititled left join heightweight using (icustay_id)

DROP MATERIALIZED VIEW IF EXISTS unititled2 CASCADE;

CREATE MATERIALIZED VIEW unititled2 AS

SELECT unititled1.subject_id, unititled1.icd9_code, unititled1.icustay_id, unititled1.los_hospital, unititled1.los_icu, unititled1.admission_age, unititled1.hospital_expire, unititled1.hadm_id, unititled1.gender, unititled1.admittime, unititled1.dischtime, unititled1.admission_type, unititled1.height_first, unititled1.weight_first, heightfirstday.height_echo FROM unititled1 left join heightfirstday using (icustay_id)

DROP MATERIALIZED VIEW IF EXISTS unititled3 CASCADE;

CREATE MATERIALIZED VIEW unititled3 AS

SELECT unititled2.subject_id, unititled2.icd9_code, unititled2.icustay_id, unititled2.los_hospital, unititled2.los_icu, unititled2.admission_age, unititled2.hospital_expire, unititled2.hadm_id, unititled2.gender, unititled2.admittime, unititled2.dischtime, unititled2.admission_type, unititled2.height_first, unititled2.weight_first, unititled2.height_echo, vi.heartrate_max, vi.sysbp_min, vi.diasbp_min, vi.meanbp_min, vi.resprate_max, vi.tempc_max, vi.spo2_min, vi.glucose_min, vi.glucose_max FROM unititled2 left join vitalsfirstday vi using (icustay_id)

DROP MATERIALIZED VIEW IF EXISTS unititled4 CASCADE;

CREATE MATERIALIZED VIEW unititled4 AS

SELECT unititled3.subject_id, unititled3.icd9_code, unititled3.icustay_id, unititled3.los_hospital, unititled3.los_icu, unititled3.admission_age, unititled3.hospital_expire, unititled3.hadm_id, unititled3.gender, unititled3.admittime, unititled3.dischtime, unititled3.admission_type, unititled3.height_first, unititled3.weight_first, unititled3.height_echo, unititled3.heartrate_max, unititled3.sysbp_min, unititled3.diasbp_min, unititled3.meanbp_min, unititled3.resprate_max, unititled3.tempc_max, unititled3.spo2_min, unititled3.glucose_min, unititled3.glucose_max, sofa.sofa, sofa.cardiovascular FROM unititled3 left join sofa using (icustay_id)

DROP MATERIALIZED VIEW IF EXISTS unititled5 CASCADE;

CREATE MATERIALIZED VIEW unititled5 AS

SELECT unititled4.subject_id, unititled4.icd9_code, unititled4.icustay_id, unititled4.los_hospital, unititled4.los_icu, unititled4.admission_age, unititled4.hospital_expire, unititled4.hadm_id, unititled4.gender, unititled4.admittime, unititled4.dischtime, unititled4.admission_type, unititled4.height_first, unititled4.weight_first, unititled4.height_echo, unititled4.heartrate_max, unititled4.sysbp_min, unititled4.diasbp_min, unititled4.meanbp_min, unititled4.resprate_max, unititled4.tempc_max, unititled4.spo2_min, unititled4.glucose_min, unititled4.glucose_max, unititled4.sofa, unititled4.cardiovascular, saps.saps FROM unititled4 left join saps using (icustay_id)

DROP MATERIALIZED VIEW IF EXISTS unititled6 CASCADE;

CREATE MATERIALIZED VIEW unititled6 AS

SELECT unititled5.subject_id, unititled5.icd9_code, unititled5.icustay_id, unititled5.los_hospital, unititled5.los_icu, unititled5.admission_age, unititled5.hospital_expire, unititled5.hadm_id, unititled5.gender, unititled5.admittime, unititled5.dischtime, unititled5.admission_type, unititled5.height_first, unititled5.weight_first, unititled5.height_echo, unititled5.heartrate_max, unititled5.sysbp_min, unititled5.diasbp_min, unititled5.meanbp_min, unititled5.resprate_max, unititled5.tempc_max, unititled5.spo2_min, unititled5.glucose_min, unititled5.glucose_max, unititled5.sofa, unititled5.cardiovascular, unititled5.saps, sapsii.sapsii FROM unititled5 left join sapsii using (icustay_id)

DROP MATERIALIZED VIEW IF EXISTS unititled7 CASCADE;

CREATE MATERIALIZED VIEW unititled7 AS

SELECT unititled6.subject_id, unititled6.icd9_code, unititled6.icustay_id, unititled6.los_hospital, unititled6.los_icu, unititled6.admission_age, unititled6.hospital_expire, unititled6.hadm_id, unititled6.gender, unititled6.admittime, unititled6.dischtime, unititled6.admission_type, unititled6.height_first, unititled6.weight_first, unititled6.height_echo, unititled6.heartrate_max, unititled6.sysbp_min, unititled6.diasbp_min, unititled6.meanbp_min, unititled6.resprate_max, unititled6.tempc_max, unititled6.spo2_min, unititled6.glucose_min, unititled6.glucose_max, unititled6.sofa, unititled6.cardiovascular, unititled6.saps, unititled6.sapsii, elixhauser_ahrq_score.elihauser_vanwalraven FROM unititled6 left join elixhauser_ahrq_score using (icustay_id)

DROP MATERIALIZED VIEW IF EXISTS unititled8 CASCADE;

CREATE MATERIALIZED VIEW unititled8 AS

SELECT unititled7.subject_id, unititled7.icd9_code, unititled7.icustay_id, unititled7.los_hospital, unititled7.los_icu, unititled7.admission_age, unititled7.hospital_expire, unititled7.hadm_id, unititled7.gender, unititled7.admittime, unititled7.dischtime, unititled7.admission_type, unititled7.height_first, unititled7.weight_first, unititled7.height_echo, unititled7.heartrate_max, unititled7.sysbp_min, unititled7.diasbp_min, unititled7.meanbp_min, unititled7.resprate_max, unititled7.tempc_max, unititled7.spo2_min, unititled7.glucose_min, unititled7.glucose_max, unititled7.sofa, unititled7.cardiovascular, unititled7.saps, unititled7.sapsii, unititled7.elihauser_vanwalraven FROM unititled7 left join elixhauser_ahrq_score using (icustay_id)

DROP MATERIALIZED VIEW IF EXISTS unititled8 CASCADE;

CREATE MATERIALIZED VIEW unititled8 AS

SELECT unititled7.subject_id, unititled7.icd9_code, unititled7.icustay_id, unititled7.los_hospital, unititled7.los_icu, unititled7.admission_age, unititled7.hospital_expire, unititled7.hadm_id, unititled7.gender, unititled7.admittime, unititled7.dischtime, unititled7.admission_type, unititled7.height_first, unititled7.weight_first, unititled7.height_echo, unititled7.heartrate_max, unititled7.sysbp_min, unititled7.diasbp_min, unititled7.meanbp_min, unititled7.resprate_max, unititled7.tempc_max, unititled7.spo2_min, unititled7.glucose_min, unititled7.glucose_max, unititled7.sofa, unititled7.cardiovascular, unititled7.saps, unititled7.sapsii, unititled7.elixhauser_vanwalraven, dobutaminedurations1.sum FROM unititled7 left join dobutaminedurations1 using (icustay_id)

DROP MATERIALIZED VIEW IF EXISTS unititled10 CASCADE;

CREATE MATERIALIZED VIEW unititled10 AS

SELECT unititled8.subject_id, unititled8.icd9_code, unititled8.icustay_id, unititled8.los_hospital, unititled8.los_icu, unititled8.admission_age, unititled8.hospital_expire, unititled8.hadm_id, unititled8.gender, unititled8.admittime, unititled8.dischtime, unititled8.admission_type, unititled8.height_first, unititled8.weight_first, unititled8.height_echo, unititled8.heartrate_max, unititled8.sysbp_min, unititled8.diasbp_min, unititled8.meanbp_min, unititled8.resprate_max, unititled8.tempc_max, unititled8.spo2_min, unititled8.glucose_min, unititled8.glucose_max, unititled8.sofa, unititled8.cardiovascular, unititled8.saps, unititled8.sapsii, unititled8.elixhauser_vanwalraven, unititled8.sum, urine.sum as urineoutputsum FROM unititled8 left join urineoutputtotal urine using (icustay_id)

DROP MATERIALIZED VIEW IF EXISTS unititled11 CASCADE;

CREATE MATERIALIZED VIEW unititled11 AS

SELECT unititled10.subject_id, unititled10.icd9_code, unititled10.icustay_id, unititled10.los_hospital, unititled10.los_icu, unititled10.admission_age, unititled10.hospital_expire, unititled10.hadm_id, unititled10.gender, unititled10.admittime, unititled10.dischtime, unititled10.admission_type, unititled10.height_first, unititled10.weight_first, unititled10.height_echo, unititled10.heartrate_max, unititled10.sysbp_min, unititled10.diasbp_min, unititled10.meanbp_min, unititled10.resprate_max, unititled10.tempc_max, unititled10.spo2_min, unititled10.glucose_min, unititled10.glucose_max, unititled10.sofa, unititled10.cardiovascular, unititled10.saps, unititled10.sapsii, unititled10.elixhauser_vanwalraven, unititled10.sum, unititled10.urineoutputsum, inputtotal.inputtotal FROM unititled10 left join inputtotal using (icustay_id)

DROP MATERIALIZED VIEW IF EXISTS unititled12 CASCADE;

CREATE MATERIALIZED VIEW unititled12 AS

SELECT unititled11.subject_id, unititled11.icd9_code, unititled11.icustay_id, unititled11.los_hospital, unititled11.los_icu, unititled11.admission_age, unititled11.hospital_expire, unititled11.hadm_id, unititled11.gender, unititled11.admittime, unititled11.dischtime, unititled11.admission_type, unititled11.height_first, unititled11.weight_first, unititled11.height_echo, unititled11.heartrate_max, unititled11.sysbp_min, unititled11.diasbp_min, unititled11.meanbp_min, unititled11.resprate_max, unititled11.tempc_max, unititled11.spo2_min, unititled11.glucose_min, unititled11.glucose_max, unititled11.sofa, unititled11.cardiovascular, unititled11.saps, unititled11.sapsii, unititled11.elixhauser_vanwalraven, unititled11.sum, unititled11.urineoutputsum, unititled11.inputtotal, elix.congestive_heart_failure, elix.cardiac_arrhythmias, elix.valvular_disease, elix.pulmonary_circulation, elix.peripheral_vascular, elix.hypertension, elix.paralysis, elix.other_neurological, elix.chronic_pulmonary, elix.diabetes_uncomplicated, elix.diabetes_complicated, elix.hypothyroidism, elix.renal_failure, elix.liver_disease, elix.peptic_ulcer, elix. Lymphoma, elix. metastatic_cancer, elix.solid_tumor, elix.coagulopathy, elix.obesity, elix.weight_loss, elix.fluid_electrolyte, elix.blood_loss_anemia, elix.deficiency_anemias, elix.alcohol_abuse, elix. drug_abuse, elix.psychoses, elix.depression FROM unititled11 left join elixhauser_quan elix using (icustay_id)

DROP MATERIALIZED VIEW IF EXISTS unititled13 CASCADE; （先不用，转至14）

CREATE MATERIALIZED VIEW unititled13 AS

SELECT unititled12.subject_id, unititled12.icd9_code, unititled12.icustay_id, unititled12.los_hospital, unititled12.los_icu, unititled12.admission_age, unititled12.hospital_expire, unititled12.hadm_id, unititled12.gender, unititled12.admittime, unititled12.dischtime, unititled12.admission_type, unititled12.height_first, unititled12.weight_first, unititled12.height_echo, unititled12.heartrate_max, unititled12.sysbp_min, unititled12.diasbp_min, unititled12.meanbp_min, unititled12.resprate_max, unititled12.tempc_max, unititled12.spo2_min, unititled12.glucose_min, unititled12.glucose_max, unititled12.sofa, unititled12.cardiovascular, unititled12.saps, unititled12.sapsii, unititled12.elixhauser_vanwalraven, unititled12.sum, unititled12.urineoutputsum, unititled12.inputtotal, unititled12.congestive_heart_failure, unititled12.cardiac_arrhythmias, unititled12.valvular_disease, unititled12.pulmonary_circulation, unititled12.peripheral_vascular, unititled12.hypertension, unititled12.paralysis, unititled12.other_neurological, unititled12.chronic_pulmonary, unititled12.diabetes_uncomplicated, unititled12.diabetes_complicated, unititled12.hypothyroidism, unititled12.renal_failure, unititled12.liver_disease, unititled12.peptic_ulcer, unititled12. Lymphoma, unititled12. metastatic_cancer, unititled12.solid_tumor, unititled12.coagulopathy, unititled12.obesity, unititled12.weight_loss, unititled12.fluid_electrolyte, unititled12.blood_loss_anemia, unititled12.deficiency_anemias, unititled12.alcohol_abuse, unititled12. drug_abuse, unititled12.psychoses, unititled12.depression, micro.org_name FROM unititled12 left join microbiologyevents micro using (hadm_id)

DROP MATERIALIZED VIEW IF EXISTS unititled14 CASCADE;

CREATE MATERIALIZED VIEW unititled14 AS

SELECT unititled12.subject_id, unititled12.icd9_code, unititled12.icustay_id, unititled12.los_hospital, unititled12.los_icu, unititled12.admission_age, unititled12.hospital_expire, unititled12.hadm_id, unititled12.gender, unititled12.admittime, unititled12.dischtime, unititled12.admission_type, unititled12.height_first, unititled12.weight_first, unititled12.height_echo, unititled12.heartrate_max, unititled12.sysbp_min, unititled12.diasbp_min, unititled12.meanbp_min, unititled12.resprate_max, unititled12.tempc_max, unititled12.spo2_min, unititled12.glucose_min, unititled12.glucose_max, unititled12.sofa, unititled12.cardiovascular, unititled12.saps, unititled12.sapsii, unititled12.elixhauser_vanwalraven, unititled12.sum, unititled12.urineoutputsum, unititled12.inputtotal, unititled12.congestive_heart_failure, unititled12.cardiac_arrhythmias, unititled12.valvular_disease, unititled12.pulmonary_circulation, unititled12.peripheral_vascular, unititled12.hypertension, unititled12.paralysis, unititled12.other_neurological, unititled12.chronic_pulmonary, unititled12.diabetes_uncomplicated, unititled12.diabetes_complicated, unititled12.hypothyroidism, unititled12.renal_failure, unititled12.liver_disease, unititled12.peptic_ulcer, unititled12. Lymphoma, unititled12. metastatic_cancer, unititled12.solid_tumor, unititled12.coagulopathy, unititled12.obesity, unititled12.weight_loss, unititled12.fluid_electrolyte, unititled12.blood_loss_anemia, unititled12.deficiency_anemias, unititled12.alcohol_abuse, unititled12. drug_abuse, unititled12.psychoses, unititled12.depression, rrtfirstday.rrt as rrtfirstday FROM unititled12 left join rrtfirstday using (icustay_id)

DROP MATERIALIZED VIEW IF EXISTS unititled15 CASCADE;

CREATE MATERIALIZED VIEW unititled15 AS

SELECT unititled14.subject_id, unititled14.icd9_code, unititled14.icustay_id, unititled14.los_hospital, unititled14.los_icu, unititled14.admission_age, unititled14.hospital_expire, unititled14.hadm_id, unititled14.gender, unititled14.admittime, unititled14.dischtime, unititled14.admission_type, unititled14.height_first, unititled14.weight_first, unititled14.height_echo, unititled14.heartrate_max, unititled14.sysbp_min, unititled14.diasbp_min, unititled14.meanbp_min, unititled14.resprate_max, unititled14.tempc_max, unititled14.spo2_min, unititled14.glucose_min, unititled14.glucose_max, unititled14.sofa, unititled14.cardiovascular, unititled14.saps, unititled14.sapsii, unititled14.elixhauser_vanwalraven, unititled14.sum, unititled14.urineoutputsum, unititled14.inputtotal, unititled14.congestive_heart_failure, unititled14.cardiac_arrhythmias, unititled14.valvular_disease, unititled14.pulmonary_circulation, unititled14.peripheral_vascular, unititled14.hypertension, unititled14.paralysis, unititled14.other_neurological, unititled14.chronic_pulmonary, unititled14.diabetes_uncomplicated, unititled14.diabetes_complicated, unititled14.hypothyroidism, unititled14.renal_failure, unititled14.liver_disease, unititled14.peptic_ulcer, unititled14. Lymphoma, unititled14. metastatic_cancer, unititled14.solid_tumor, unititled14.coagulopathy, unititled14.obesity, unititled14.weight_loss, unititled14.fluid_electrolyte, unititled14.blood_loss_anemia, unititled14.deficiency_anemias, unititled14.alcohol_abuse, unititled14. drug_abuse, unititled14.psychoses, unititled14.depression, unititled14.rrtfirstday, ventfirstday.vent as ventfirstday FROM unititled14 left join ventfirstday using (icustay_id)

DROP MATERIALIZED VIEW IF EXISTS unititled16 CASCADE;

CREATE MATERIALIZED VIEW unititled16 AS

SELECT unititled15.subject_id, unititled15.icd9_code, unititled15.icustay_id, unititled15.los_hospital, unititled15.los_icu, unititled15.admission_age, unititled15.hospital_expire, unititled15.hadm_id, unititled15.gender, unititled15.admittime, unititled15.dischtime, unititled15.admission_type, unititled15.height_first, unititled15.weight_first, unititled15.height_echo, unititled15.heartrate_max, unititled15.sysbp_min, unititled15.diasbp_min, unititled15.meanbp_min, unititled15.resprate_max, unititled15.tempc_max, unititled15.spo2_min, unititled15.glucose_min, unititled15.glucose_max, unititled15.sofa, unititled15.cardiovascular, unititled15.saps, unititled15.sapsii, unititled15.elixhauser_vanwalraven, unititled15.sum, unititled15.urineoutputsum, unititled15.inputtotal, unititled15.congestive_heart_failure, unititled15.cardiac_arrhythmias, unititled15.valvular_disease, unititled15.pulmonary_circulation, unititled15.peripheral_vascular, unititled15.hypertension, unititled15.paralysis, unititled15.other_neurological, unititled15.chronic_pulmonary, unititled15.diabetes_uncomplicated, unititled15.diabetes_complicated, unititled15.hypothyroidism, unititled15.renal_failure, unititled15.liver_disease, unititled15.peptic_ulcer, unititled15. Lymphoma, unititled15. metastatic_cancer, unititled15.solid_tumor, unititled15.coagulopathy, unititled15.obesity, unititled15.weight_loss, unititled15.fluid_electrolyte, unititled15.blood_loss_anemia, unititled15.deficiency_anemias, unititled15.alcohol_abuse, unititled15. drug_abuse, unititled15.psychoses, unititled15.depression, unititled15.rrtfirstday, unititled15.ventfirstday, labevents."value" FROM unititled15 left join labevents using (subject_id) where labevents.itemid=50813 and labevents.charttime between unititled15.admittime and unititled15.admittime+6 hour

DROP MATERIALIZED VIEW IF EXISTS unititled17 CASCADE;

CREATE MATERIALIZED VIEW unititled17 AS

SELECT unititled16.subject_id, unititled16.icd9_code, unititled16.icustay_id, unititled16.los_hospital, unititled16.los_icu, unititled16.admission_age, unititled16.hospital_expire, unititled16.hadm_id, unititled16.gender, unititled16.admittime, unititled16.dischtime, unititled16.admission_type, unititled16.height_first, unititled16.weight_first, unititled16.height_echo, unititled16.heartrate_max, unititled16.sysbp_min, unititled16.diasbp_min, unititled16.meanbp_min, unititled16.resprate_max, unititled16.tempc_max, unititled16.spo2_min, unititled16.glucose_min, unititled16.glucose_max, unititled16.sofa, unititled16.cardiovascular, unititled16.saps, unititled16.sapsii, unititled16.elixhauser_vanwalraven, unititled16.sum, unititled16.urineoutputsum, unititled16.inputtotal, unititled16.congestive_heart_failure, unititled16.cardiac_arrhythmias, unititled16.valvular_disease, unititled16.pulmonary_circulation, unititled16.peripheral_vascular, unititled16.hypertension, unititled16.paralysis, unititled16.other_neurological, unititled16.chronic_pulmonary, unititled16.diabetes_uncomplicated, unititled16.diabetes_complicated, unititled16.hypothyroidism, unititled16.renal_failure, unititled16.liver_disease, unititled16.peptic_ulcer, unititled16. Lymphoma, unititled16. metastatic_cancer, unititled16.solid_tumor, unititled16.coagulopathy, unititled16.obesity, unititled16.weight_loss, unititled16.fluid_electrolyte, unititled16.blood_loss_anemia, unititled16.deficiency_anemias, unititled16.alcohol_abuse, unititled16. drug_abuse, unititled16.psychoses, unititled16.depression, unititled16.rrtfirstday, unititled16.ventfirstday, unititled16.lactatevalue, min(unititled16.charttime) FROM unititled16 group by unititled16.subject_id

DROP MATERIALIZED VIEW IF EXISTS unititled18 CASCADE;

CREATE MATERIALIZED VIEW unititled18 AS

SELECT unititled17.subject_id, unititled17.icd9_code, unititled17.icustay_id, unititled17.los_hospital, unititled17.los_icu, unititled17.admission_age, unititled17.hospital_expire, unititled17.hadm_id, unititled17.gender, unititled17.admittime, unititled17.dischtime, unititled17.admission_type, unititled17.height_first, unititled17.weight_first, unititled17.height_echo, unititled17.heartrate_max, unititled17.sysbp_min, unititled17.diasbp_min, unititled17.meanbp_min, unititled17.resprate_max, unititled17.tempc_max, unititled17.spo2_min, unititled17.glucose_min, unititled17.glucose_max, unititled17.sofa, unititled17.cardiovascular, unititled17.saps, unititled17.sapsii, unititled17.elixhauser_vanwalraven, unititled17.sum, unititled17.urineoutputsum, unititled17.inputtotal, unititled17.congestive_heart_failure, unititled17.cardiac_arrhythmias, unititled17.valvular_disease, unititled17.pulmonary_circulation, unititled17.peripheral_vascular, unititled17.hypertension, unititled17.paralysis, unititled17.other_neurological, unititled17.chronic_pulmonary, unititled17.diabetes_uncomplicated, unititled17.diabetes_complicated, unititled17.hypothyroidism, unititled17.renal_failure, unititled17.liver_disease, unititled17.peptic_ulcer, unititled17. Lymphoma, unititled17. metastatic_cancer, unititled17.solid_tumor, unititled17.coagulopathy, unititled17.obesity, unititled17.weight_loss, unititled17.fluid_electrolyte, unititled17.blood_loss_anemia, unititled17.deficiency_anemias, unititled17.alcohol_abuse, unititled17. drug_abuse, unititled17.psychoses, unititled17.depression, unititled17.rrtfirstday, unititled17.ventfirstday, unititled17.lactatevalue, unititled17.mincharttime, norepinephrinedurations1.sum as norepinephrinedurationsum FROM unititled17 left join norepinephrinedurations1 using(icustay_id)

DROP MATERIALIZED VIEW IF EXISTS unititled19 CASCADE;

CREATE MATERIALIZED VIEW unititled19 AS

SELECT unititled18.subject_id, unititled18.icd9_code, unititled18.icustay_id, unititled18.los_hospital, unititled18.los_icu, unititled18.admission_age, unititled18.hospital_expire, unititled18.hadm_id, unititled18.gender, unititled18.admittime, unititled18.dischtime, unititled18.admission_type, unititled18.height_first, unititled18.weight_first, unititled18.height_echo, unititled18.heartrate_max, unititled18.sysbp_min, unititled18.diasbp_min, unititled18.meanbp_min, unititled18.resprate_max, unititled18.tempc_max, unititled18.spo2_min, unititled18.glucose_min, unititled18.glucose_max, unititled18.sofa, unititled18.cardiovascular, unititled18.saps, unititled18.sapsii, unititled18.elixhauser_vanwalraven, unititled18.sum, unititled18.urineoutputsum, unititled18.inputtotal, unititled18.congestive_heart_failure, unititled18.cardiac_arrhythmias, unititled18.valvular_disease, unititled18.pulmonary_circulation, unititled18.peripheral_vascular, unititled18.hypertension, unititled18.paralysis, unititled18.other_neurological, unititled18.chronic_pulmonary, unititled18.diabetes_uncomplicated, unititled18.diabetes_complicated, unititled18.hypothyroidism, unititled18.renal_failure, unititled18.liver_disease, unititled18.peptic_ulcer, unititled18. Lymphoma, unititled18. metastatic_cancer, unititled18.solid_tumor, unititled18.coagulopathy, unititled18.obesity, unititled18.weight_loss, unititled18.fluid_electrolyte, unititled18.blood_loss_anemia, unititled18.deficiency_anemias, unititled18.alcohol_abuse, unititled18. drug_abuse, unititled18.psychoses, unititled18.depression, unititled18.rrtfirstday, unititled18.ventfirstday, unititled18.lactatevalue, unititled18.mincharttime, unititled18.norepinephrinedurationsum, crrtduration1.sum as crrtdurationsum FROM unititled18 left join crrtduration1 using(icustay_id)

DROP MATERIALIZED VIEW IF EXISTS unititled20 CASCADE;

CREATE MATERIALIZED VIEW unititled20 AS

SELECT unititled19.subject_id, unititled19.icd9_code, unititled19.icustay_id, unititled19.los_hospital, unititled19.los_icu, unititled19.admission_age, unititled19.hospital_expire, unititled19.hadm_id, unititled19.gender, unititled19.admittime, unititled19.dischtime, unititled19.admission_type, unititled19.height_first, unititled19.weight_first, unititled19.height_echo, unititled19.heartrate_max, unititled19.sysbp_min, unititled19.diasbp_min, unititled19.meanbp_min, unititled19.resprate_max, unititled19.tempc_max, unititled19.spo2_min, unititled19.glucose_min, unititled19.glucose_max, unititled19.sofa, unititled19.cardiovascular, unititled19.saps, unititled19.sapsii, unititled19.elixhauser_vanwalraven, unititled19.sum, unititled19.urineoutputsum, unititled19.inputtotal, unititled19.congestive_heart_failure, unititled19.cardiac_arrhythmias, unititled19.valvular_disease, unititled19.pulmonary_circulation, unititled19.peripheral_vascular, unititled19.hypertension, unititled19.paralysis, unititled19.other_neurological, unititled19.chronic_pulmonary, unititled19.diabetes_uncomplicated, unititled19.diabetes_complicated, unititled19.hypothyroidism, unititled19.renal_failure, unititled19.liver_disease, unititled19.peptic_ulcer, unititled19. Lymphoma, unititled19. metastatic_cancer, unititled19.solid_tumor, unititled19.coagulopathy, unititled19.obesity, unititled19.weight_loss, unititled19.fluid_electrolyte, unititled19.blood_loss_anemia, unititled19.deficiency_anemias, unititled19.alcohol_abuse, unititled19. drug_abuse, unititled19.psychoses, unititled19.depression, unititled19.rrtfirstday, unititled19.ventfirstday, unititled19.lactatevalue, unititled19.mincharttime, unititled19.norepinephrinedurationsum, unititled19. crrtdurationsum,ventilationduration1.sum as ventilationdurationsum FROM unititled19 left join ventilationduration1 using(icustay_id)

DROP MATERIALIZED VIEW IF EXISTS unititled21 CASCADE;

CREATE MATERIALIZED VIEW unititled21 AS

SELECT unititled20.subject_id, unititled20.icd9_code, unititled20.icustay_id, unititled20.los_hospital, unititled20.los_icu, unititled20.admission_age, unititled20.hospital_expire, unititled20.hadm_id, unititled20.gender, unititled20.admittime, unititled20.dischtime, unititled20.admission_type, unititled20.height_first, unititled20.weight_first, unititled20.height_echo, unititled20.heartrate_max, unititled20.sysbp_min, unititled20.diasbp_min, unititled20.meanbp_min, unititled20.resprate_max, unititled20.tempc_max, unititled20.spo2_min, unititled20.glucose_min, unititled20.glucose_max, unititled20.sofa, unititled20.cardiovascular, unititled20.saps, unititled20.sapsii, unititled20.elixhauser_vanwalraven, unititled20.sum, unititled20.urineoutputsum, unititled20.inputtotal, unititled20.congestive_heart_failure, unititled20.cardiac_arrhythmias, unititled20.valvular_disease, unititled20.pulmonary_circulation, unititled20.peripheral_vascular, unititled20.hypertension, unititled20.paralysis, unititled20.other_neurological, unititled20.chronic_pulmonary, unititled20.diabetes_uncomplicated, unititled20.diabetes_complicated, unititled20.hypothyroidism, unititled20.renal_failure, unititled20.liver_disease, unititled20.peptic_ulcer, unititled20. Lymphoma, unititled20. metastatic_cancer, unititled20.solid_tumor, unititled20.coagulopathy, unititled20.obesity, unititled20.weight_loss, unititled20.fluid_electrolyte, unititled20.blood_loss_anemia, unititled20.deficiency_anemias, unititled20.alcohol_abuse, unititled20. drug_abuse, unititled20.psychoses, unititled20.depression, unititled20.rrtfirstday, unititled20.ventfirstday, unititled20.lactatevalue, unititled20.mincharttime, unititled20.norepinephrinedurationsum, unititled20. crrtdurationsum, unititled20.ventilationdurationsum, milrinonedurations.duration_hours as milrinoneduration FROM unititled20 left join milrinonedurations using(icustay_id)

DROP MATERIALIZED VIEW IF EXISTS unititled22 CASCADE;

CREATE MATERIALIZED VIEW unititled22 AS

SELECT unititled21.subject_id, unititled21.icd9_code, unititled21.icustay_id, unititled21.los_hospital, unititled21.los_icu, unititled21.admission_age, unititled21.hospital_expire, unititled21.hadm_id, unititled21.gender, unititled21.admittime, unititled21.dischtime, unititled21.admission_type, unititled21.height_first, unititled21.weight_first, unititled21.height_echo, unititled21.heartrate_max, unititled21.sysbp_min, unititled21.diasbp_min, unititled21.meanbp_min, unititled21.resprate_max, unititled21.tempc_max, unititled21.spo2_min, unititled21.glucose_min, unititled21.glucose_max, unititled21.sofa, unititled21.cardiovascular, unititled21.saps, unititled21.sapsii, unititled21.elixhauser_vanwalraven, unititled21.sum, unititled21.urineoutputsum, unititled21.inputtotal, unititled21.congestive_heart_failure, unititled21.cardiac_arrhythmias, unititled21.valvular_disease, unititled21.pulmonary_circulation, unititled21.peripheral_vascular, unititled21.hypertension, unititled21.paralysis, unititled21.other_neurological, unititled21.chronic_pulmonary, unititled21.diabetes_uncomplicated, unititled21.diabetes_complicated, unititled21.hypothyroidism, unititled21.renal_failure, unititled21.liver_disease, unititled21.peptic_ulcer, unititled21. Lymphoma, unititled21. metastatic_cancer, unititled21.solid_tumor, unititled21.coagulopathy, unititled21.obesity, unititled21.weight_loss, unititled21.fluid_electrolyte, unititled21.blood_loss_anemia, unititled21.deficiency_anemias, unititled21.alcohol_abuse, unititled21. drug_abuse, unititled21.psychoses, unititled21.depression, unititled21.rrtfirstday, unititled21.ventfirstday, unititled21.lactatevalue, unititled21.mincharttime, unititled21.norepinephrinedurationsum, unititled21. crrtdurationsum, unititled21.ventilationdurationsum, unititled21.milrinoneduration, micro.org_name FROM unititled21 left join microbiologyevents micro using(hadm_id)

CREATE MATERIALIZED VIEW unititled23 AS

SELECT unititled22.subject_id, unititled22.icd9_code, unititled22.icustay_id, unititled22.los_hospital, unititled22.los_icu, unititled22.admission_age, unititled22.hospital_expire, unititled22.hadm_id, unititled22.gender, unititled22.admittime, unititled22.dischtime, unititled22.admission_type, unititled22.height_first, unititled22.weight_first, unititled22.height_echo, unititled22.heartrate_max, unititled22.sysbp_min, unititled22.diasbp_min, unititled22.meanbp_min, unititled22.resprate_max, unititled22.tempc_max, unititled22.spo2_min, unititled22.glucose_min, unititled22.glucose_max, unititled22.sofa, unititled22.cardiovascular, unititled22.saps, unititled22.sapsii, unititled22.elixhauser_vanwalraven, unititled22.sum, unititled22.urineoutputsum, unititled22.inputtotal, unititled22.congestive_heart_failure, unititled22.cardiac_arrhythmias, unititled22.valvular_disease, unititled22.pulmonary_circulation, unititled22.peripheral_vascular, unititled22.hypertension, unititled22.paralysis, unititled22.other_neurological, unititled22.chronic_pulmonary, unititled22.diabetes_uncomplicated, unititled22.diabetes_complicated, unititled22.hypothyroidism, unititled22.renal_failure, unititled22.liver_disease, unititled22.peptic_ulcer, unititled22. Lymphoma, unititled22. metastatic_cancer, unititled22.solid_tumor, unititled22.coagulopathy, unititled22.obesity, unititled22.weight_loss, unititled22.fluid_electrolyte, unititled22.blood_loss_anemia, unititled22.deficiency_anemias, unititled22.alcohol_abuse, unititled22. drug_abuse, unititled22.psychoses, unititled22.depression, unititled22.rrtfirstday, unititled22.ventfirstday, unititled22.lactatevalue, unititled22.mincharttime, unititled22.norepinephrinedurationsum, unititled22. crrtdurationsum, unititled22.ventilationdurationsum, unititled22.milrinoneduration, unititled22.org_name, echodata.height FROM unititled22 left join echodata using(hadm_id)
